# Diagnostic accuracy of ChatGPT for 12-lead ECG-based localisation of ventricular ectopic foci prior to catheter ablation

**DOI:** 10.3389/fmed.2025.1685419

**Published:** 2026-01-12

**Authors:** Kadri Murat Gürses, Hüseyin Tezcan, Muhammed Ulvi Yalçın, Halil Özalp, Abdullah Tunçez, Yasin Özen

**Affiliations:** Department of Cardiology, Selcuk University Medical Faculty Hospital, Konya, Türkiye

**Keywords:** ChatGPT, ventricular ectopy, catheter ablation, electrocardiogram, artificial intelligence

## Abstract

**Background:**

Precise pre-procedural localisation of ventricular ectopic (VE) foci shortens mapping time, reduces fluoroscopy, and improves ablation success. Large language models such as ChatGPT offer instant, free-text clinical support; however, their accuracy in ECG-based VE localisation is unknown.

**Methods:**

In this single-centre pilot study, we assessed the diagnostic accuracy of ChatGPT in 50 consecutive adults (average age: 43 ± 14 years; 58% women) scheduled for first-time VE ablation. ChatGPT served as the index test, and invasive electroanatomical mapping during the ablation served as the reference standard. A blinded electrophysiologist converted each index 12-lead ECG into a structured textual description of QRS morphology. ChatGPT-4o (temperature 0.2) was then tasked with assigning one of five anatomical origins (RVOT, LVOT, papillary muscle, fascicular, and epicardial). Predictions were compared with electro-anatomical mapping during catheter ablation, and agreement was measured using Cohen’s *κ* (κ).

**Results:**

Electro-anatomical mapping identified 30 RVOT, 11 LVOT, 4 papillary, 1 fascicular, and 4 epicardial foci. ChatGPT correctly localised 17/50 cases (34%), yielding an overall Cohen’s κ of −0.02 (95% CI –0.18 to 0.14). Sensitivity/specificity was 40%/55% for the RVOT and 36%/62% for the LVOT; no fascicular or epicardial origins were correctly predicted. The performance of ChatGPT did not differ based on the presence of structural heart disease (*p* = 0.43). The duration of the procedure and the acute ablation success rate (96%) were unaffected by the accuracy of ChatGPT.

**Conclusion:**

Freetext querying of ChatGPT failed to provide clinically meaningful VE localisation, performing no better than chance and markedly below published ECG-based algorithms. This likely reflects the model’s lack of domain-specific training and its reliance on purely text-based reasoning without direct access to ECG signals. Current general-purpose language models should not be relied upon for procedural planning in VE ablation; future work must integrate multimodal training and domain-specific optimisation before LLMs can augment electrophysiology practice.

## Introduction

Premature ventricular complexes (PVCs) and other forms of idiopathic ventricular ectopy (VE) are frequently ablated when they are symptomatic, highly prevalent, or responsible for triggering tachycardia or cardiomyopathy. Precise *a priori* localisation of the arrhythmic focus assists the operator in selecting the most appropriate optimal venous or arterial access, catheter type, and ablation strategy, thereby shortening the procedure and fluoroscopy times. Conventional localisation relies on visual inspection of 12-lead electrocardiograms (ECGs) using several stepwise algorithms. Meta-analyses consistently show only moderate accuracy (≈55–70%) in distinguishing right from left-sided outflow tract PVCs and identifying non-outflow foci such as papillary muscles or the left ventricular (LV) summit (1, 2). In particular, papillary-muscle and epicardial VEs often defy surface ECG prediction and are associated with lower rates of acute and long-term ablation success (3, 4).

Artificial intelligence (AI) approaches have begun to fill this gap. Deep-learning models trained on thousands of digital ECGs can now localise VE exits with millimetre-level precision, especially for anatomically complex regions such as the LV summit (5, 6). However, these systems require large, labelled ECG datasets and custom software pipelines, limiting immediate clinical deployment. Large language models (LLMs), such as ChatGPT (OpenAI, San Francisco, CA), offer a radically different paradigm: they accept plain-text descriptions and generate probabilistic answers without additional coding. Early exploratory work suggests that ChatGPT can reason over structured clinical vignettes and provide guideline-concordant recommendations in cardiology and electrophysiology; however, its ability to interpret detailed ECG morphology has not been prospectively tested (7).

We therefore designed a single-centre, prospective study to quantitatively compare ChatGPT-derived localisation of VE foci—obtained from text-based ECG descriptions—with the gold standard of invasive electro-anatomical mapping during catheter ablation. We hypothesised that ChatGPT would outperform historical ECG algorithms and achieve a clinically acceptable agreement (*κ* ≥ 0.6) with electrophysiology study (EPS) results.

## Materials and methods

This was a single-centre, exploratory diagnostic accuracy pilot study, designed and reported with reference to STARD guidelines, conducted at the Selçuk University Faculty of Medicine Arrhythmia Centre, a tertiary referral institution equipped with CARTO 3 (Biosense Webster), EnSite Precision (Abbott), and Rhythmia (Boston Scientific) mapping platforms. The enrolment window was 1 January – 30 June 2025 (6 months).

### Study population

Fifty consecutive adults (≥18 years) scheduled for first-time catheter ablation for frequent or symptomatic VE were screened. The inclusion criteria were as follows:

- ≥5% PVC burden or symptomatic monomorphic VE documented on 12-lead ECG or ambulatory monitor
- Availability of a high-quality 12-lead ECG of the index morphology recorded ≤30 days before ablation
- Written informed consent

The exclusion criteria were as follows:

- Multifocal VE or polymorphic ventricular tachycardia
- Failure to induce/identify the clinical VE during EPS
- Uninterpretable ECG due to artefact or missing leads
- Previous ablation for the same morphology

Approval was obtained from the Selçuk University Faculty of Medicine Local Ethics Committee (Decision No. 2025/178), and all procedures conformed to the ethical standards of the Declaration of Helsinki. Written informed consent was obtained from each participant before any study procedures were undertaken.

### Index test and reference standard

ChatGPT-4o (temperature 0.2) generated a single forced-choice localisation (RVOT, LVOT, papillary muscle, fascicular/His–Purkinje, or epicardial/LV summit) from a blinded, structured textual ECG description for each case. Invasive electroanatomical mapping during catheter ablation was defined as the true focus (earliest activation ≥ 20 ms pre-QRS and/or ≥ 11/12 pacemap match). Operators were blinded to the ChatGPT outputs during the procedures. The electrophysiologist preparing textual ECG descriptors and the ChatGPT querying were blinded to the electroanatomical results, and mapping operators were blinded to the ChatGPT predictions.

### ECG acquisition and structured description

Standard 12-lead ECGs (25 mm s^−1^, 10 mm mV^−1^) were exported as PDF and anonymised. One senior electrophysiologist (blinded to EPS results) generated a concise, rule-based textual description for each PVC morphology, including:

- frontal QRS axis (inferior, superior, rightward, leftward)
- bundle-branch-block pattern (RBB-like vs. LBB-like)
- earliest precordial transition (leads V₁–V₆)
- presence of QRS notching, slurring, or pseudodelta
- limb-lead concordance and other salient features

This approach mirrors the published ECG algorithms and provides structured input while remaining agnostic to any proprietary data format (1). To enhance transparency and reproducibility, representative examples of complete textual descriptions, together with the exact ChatGPT prompt, are provided in Supplementary Table S1.

### Human ECG localisation comparator

An experienced electrophysiologist (EP), distinct from the ECG descriptor author and blinded to both ChatGPT outputs and electroanatomic mapping (EAM) results, independently reviewed each index 12-lead ECG and assigned a single most likely origin among five prespecified categories (RVOT, LVOT, papillary muscle, fascicular/His–Purkinje, or epicardial/LV summit) using standard surface ECG criteria. For comparative statistics, overall and class-wise diagnostic metrics were calculated against EAM. Paired accuracy between EP and ChatGPT was compared using McNemar’s test; 95% confidence intervals (CIs) for Δaccuracy were estimated using the Wilson-based transformation of discordant pairs. Agreement with EAM was quantified using unweighted Cohen’s *κ* (95% CIs). We also report balanced accuracy (macro recall) and macro F1. Case-level cross-classification is provided in Table 1 and Supplementary Table S1.

**Table 1:** EP cross-classification (actual vs. EP-predicted).

Actual/Predicted	RVOT	LVOT	Papillary	Fascicular	Epicardial	Row total
RVOT (*n* = 30)	**23**	5	1	0	1	30
LVOT (*n* = 11)	3	**8**	0	0	0	11
Papillary (*n* = 4)	1	1	**2**	0	0	4
Fascicular (*n* = 1)	0	0	0	**1**	0	1
Epicardial (*n* = 4)	1	1	0	0	**2**	4
Column totals	28	15	3	1	3	50

Rows: EAM-confirmed region (actual). Columns: EP-predicted region. Totals = 50. Overall EP accuracy = 36/50 = 72%; Cohen’s κ ≈ 0.52; macro-recall ≈ 69.9%; and macro-F1 ≈ 0.71. Outflow vs. non-outflow (EP): TP = 39, TN = 5, FP = 4, FN = 2 → accuracy = 88%, sensitivity = 95.1%, specificity = 55.6%, PPV = 90.7%, NPV = 71.4%. Bold values indicate correct predictions and/or primary performance metrics.

### ChatGPT query protocol

Using the March-2025 ChatGPT-4o model (temperature = 0.2, max tokens = 128), the following prompt was submitted for each case:

“Based on the following 12-lead ECG features, determine the most likely anatomical origin of the ventricular ectopy: [text description]. Choose one: RVOT, LVOT, papillary muscle, fascicular/His–Purkinje, or epicardial/LV summit.” Each ECG description was submitted once, and the first valid categorical answer was taken as the index test result to reflect a realistic single-query clinical use case; we did not perform systematic repeated sampling of the same prompt.

ChatGPT’s top-ranked answer was recorded. The LLM had no access to patient identifiers or EPS data.

A deliberately low temperature (0.2) was chosen to minimise output randomness and enhance reproducibility, treating ChatGPT as a deterministic decision-support tool rather than a creative generator, consistent with prior cardiology applications that emphasise guideline-concordant and stable responses. The max-tokens limit (128) was set as a conservative upper bound to prevent excessively long free-text completions while leaving ample headroom for the short requested categorical answers; this constraint affects only the response length and does not encode any domain-specific information.

### Electrophysiological study and ablation

Procedures were performed under conscious sedation using a standard protocol consistent with current international guidelines (8). Activation and pace-mapping were guided by a three-dimensional electro-anatomical system. The site of earliest local ventricular activation (≥20 ms pre-QRS) or the best pace-map (≥11/12-lead match) was deemed the true focus. Radiofrequency energy (35–50 W) or cryothermal energy was delivered until VE was suppressed. Acute procedural success was defined as the complete elimination of the clinical morphology after a 30-min waiting period and isoprenaline provocation. In each case, the clinically accepted focus—defined by these activation/pacemapping criteria and confirmed by acute procedural success—served as the reference standard for analysis. Electroanatomical maps were not independently readjudicated by a second blinded operator, and the intra-procedural reproducibility of mapping was not formally quantified.

### Outcome measures

- Primary endpoint: Agreement between the ChatGPT-predicted region and EPS-confirmed region, expressed as unweighted Cohen’s *κ* with 95% confidence intervals.
- Secondary endpoints:
  - Sensitivity, specificity, and positive and negative predictive values for (a) outflow tract VEs (RVOT + LVOT) and (b) non-outflow VEs.
  - Subgroup analysis of patients with vs. without structural heart disease.

### Sample size calculation

Pilot data and literature reports indicate ~60% localisation accuracy for conventional ECG criteria (2). To detect an absolute 20-percentage-point improvement (to ≥80%) with *α* = 0.05 and power = 0.80 (two-sided), 46 independent observations are needed (McNemar test). Anticipating up to 10% attrition due to non-inducibility, we targeted 50 patients.

### Statistical analysis

Continuous variables were presented as mean ± SD or median (IQR), depending on normality (Shapiro–Wilk test), and compared using the *t*-test or Mann–Whitney *U*-test, as appropriate. Categorical data were expressed as counts (%) and compared using the χ^2^ test or Fisher’s exact test. For diagnostic accuracy analysis, agreement between the index test (ChatGPT) and the reference standard (electroanatomical mapping) was assessed using overall multiclass accuracy (proportion of correctly classified cases) and Cohen’s *κ* with 95% confidence intervals, treating the five anatomical categories as nominal. To account for class imbalance, we additionally report class-balanced (macro) recall (“balanced accuracy”) and macro-F1 across the five classes, together with region-wise sensitivity, specificity, positive predictive value (PPV) and negative predictive value (NPV). Confidence intervals for proportions were calculated using Wilson’s method, and Cohen’s κ was calculated as an unweighted statistic. A two-tailed *p*-value of < 0.05 indicates statistical significance. Analyses were performed using R 4.3.2 (R Foundation for Statistical Computing, Vienna, Austria).

### Data protection

All ECGs were pseudonymised using study ID codes; the key file was stored on an encrypted, access-controlled hospital server. Only aggregate, de-identified data will be published.

## Results

Fifty consecutive patients (mean age 43 ± 14 years; 29 women, 58%) fulfilled the study criteria and were analysed. As summarised in Table 2, structural heart disease (SHD) was present in 12 participants (24%), and the mean left ventricular ejection fraction was 58 ± 6%. The median pre-procedural PVC burden was 17% (IQR 12–23%), and 36% were receiving *β*-blocker therapy. Electro-anatomical mapping localised the ventricular ectopy (VE) focus to the right-ventricular outflow tract (RVOT) in 30 patients (60%), the left-ventricular outflow tract (LVOT) in 11 (22%), papillary muscles in 4 (8%), epicardial/LV-summit sites in 4 (8%), and the fascicular system in 1 (2%). Acute ablation was successful in 48 (96%) patients.

**Table 2:** Baseline clinical and procedural characteristics of the study cohort (*n* = 50).

Variable	Value
Age, years	43 ± 14
Female sex, *n* (%)	29 (58%)
Structural heart disease, *n* (%)	12 (24%)
Left-ventricular ejection fraction, %	58 ± 6
PVC burden before ablation, % (median [IQR])	17 [12–23]
β-blocker therapy pre-procedure, *n* (%)	18 (36%)
Distribution of VE foci confirmed by EPS, *n* (%):	
Right-ventricular outflow tract (RVOT)	30 (60%)
Left-ventricular outflow tract (LVOT)	11 (22%)
Papillary muscle	4 (8%)
Fascicular/His-Purkinje system	1 (2%)
Epicardial/LV-summit	4 (8%)
Acute ablation success, *n* (% of total)	48 (96%)

Values are presented as the mean ± SD unless otherwise indicated.

**Figure 1:**
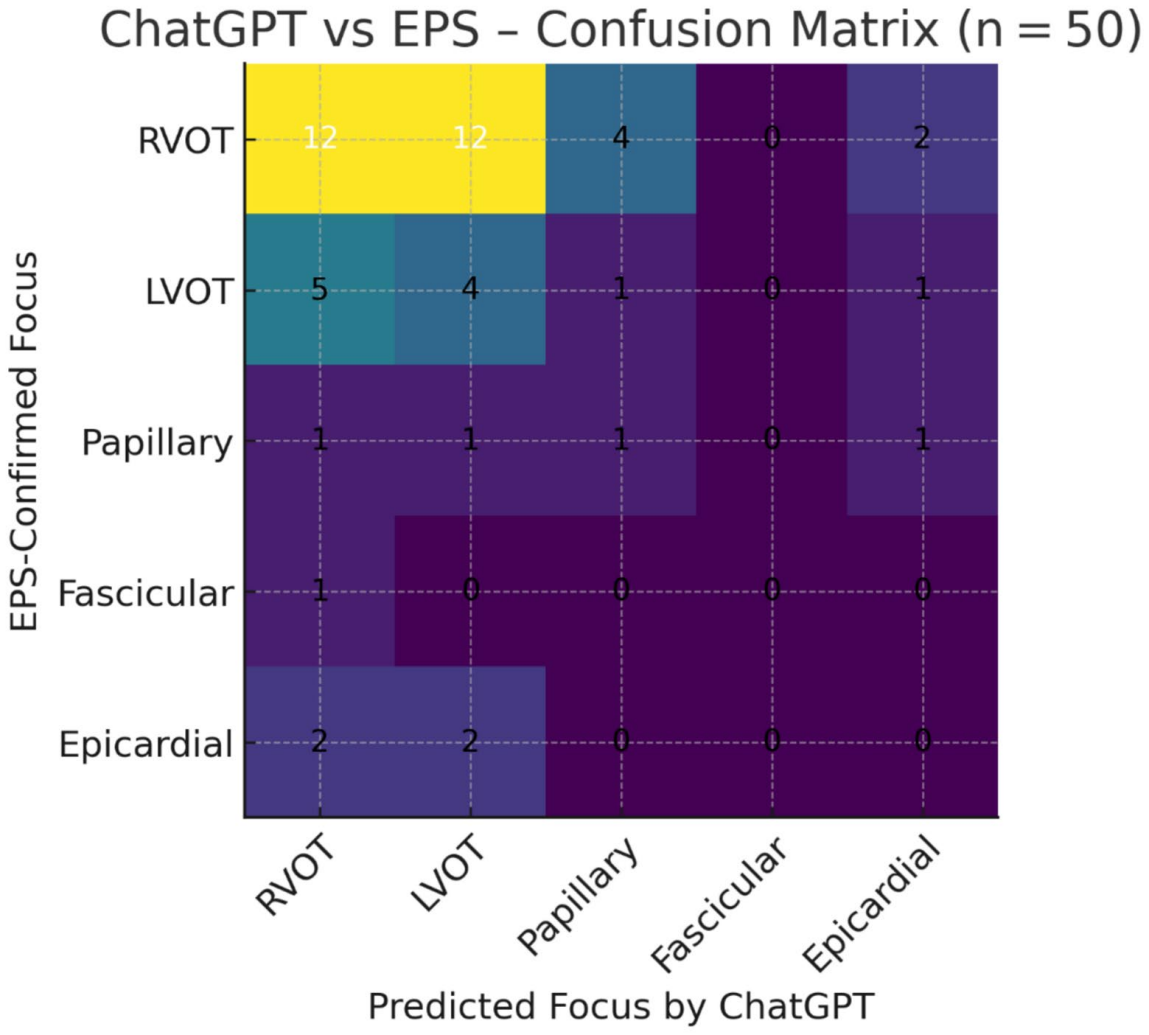
Graphical summary of ChatGPT performance. The heat map below visualises the 50-patient confusion matrix, with colour intensity proportional to the number of cases in each cell. Diagonal cells (correct predictions) are sparse, underscoring the low overall accuracy (34%) and near-random agreement (*κ* ≈ 0). Misclassifications cluster mainly between RVOT ↔ LVOT and spill over into papillary or epicardial bins—an at-a-glance illustration of the model’s systematic bias towards outflow-tract origins.

**Figure 2:**
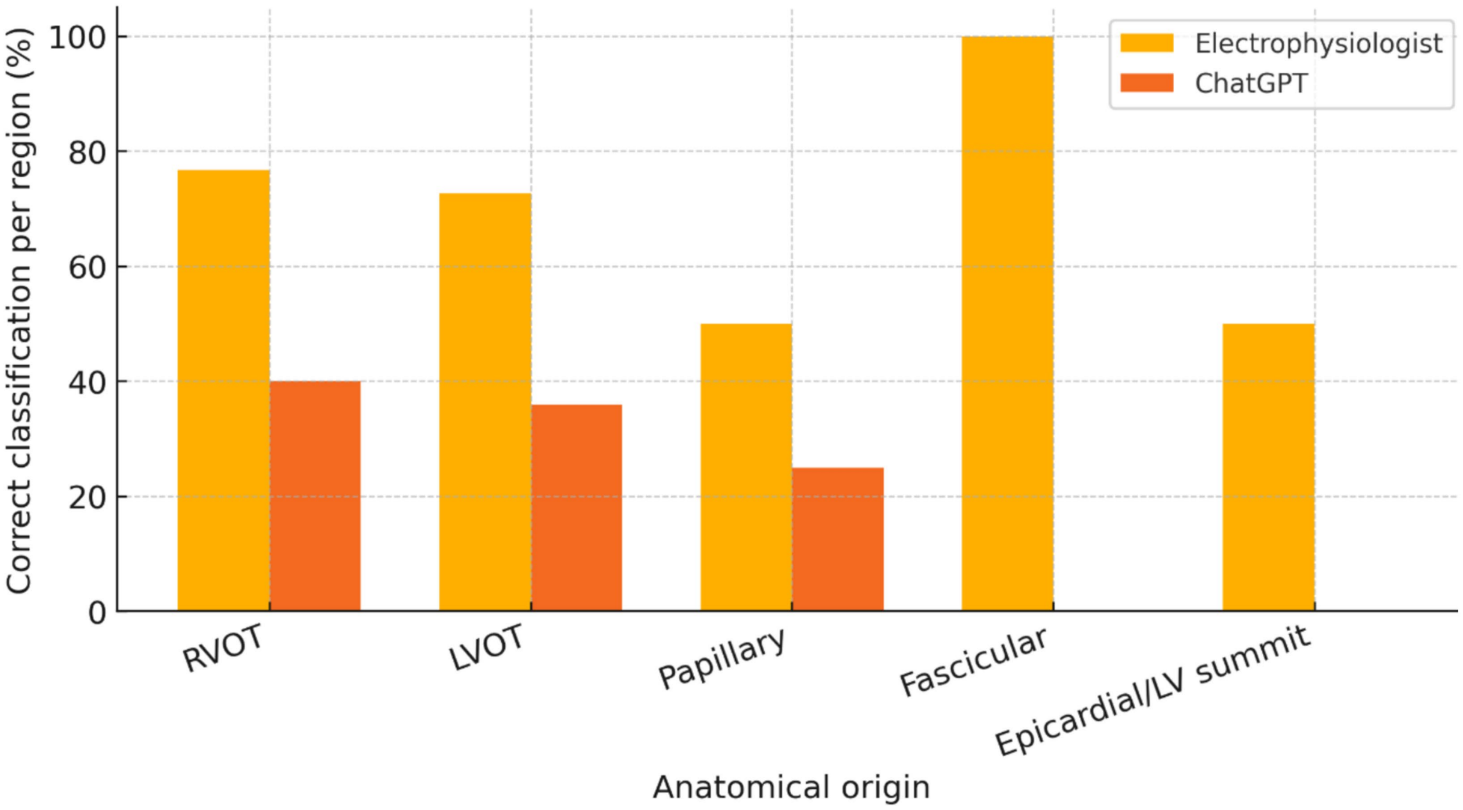
Region-wise localization performance of ChatGPT and the electrophysiologist. Bar plot showing the proportion of correctly localized ventricular ectopy (VE) foci for each electro-anatomical mapping (EAM)-confirmed region (RVOT, LVOT, papillary muscle, fascicular/His–Purkinje, or epicardial/LV summit). For each region, the left bar represents the blinded electrophysiologist (EP), and the right bar represents ChatGPT. Bar heights correspond to per-region sensitivity using EAM as the gold standard.

ChatGPT correctly identified the anatomical origin of VE in 17 of 50 patients (34%), corresponding to an overall Cohen’s *κ* of −0.02 (95% CI –0.18 to 0.14), i.e., no better than chance. Class-balanced performance was also poor: the 5-class balanced accuracy (macro-recall) was 20.2%, and the macro-F1 score was 0.19, consistent with near-random classification despite the predominance of RVOT foci. The full cross-classification of predictions versus invasive findings is provided in Table 3 and visualised in Figure 1. Region-wise correct classification rates for each anatomical origin are summarised in Figure 2. Sensitivity and specificity for RVOT localisation were 40 and 55%, respectively, and for LVOT, 36 and 62%, respectively. No correct predictions were made for fascicular or epicardial/LV-summit foci, and region-specific diagnostic metrics are detailed in Table 4. Positive predictive value exceeded 50% only for RVOT (57%), while the negative predictive value surpassed 90% for papillary, fascicular, and epicardial sites, reflecting ChatGPT’s systematic bias towards outflow-tract diagnoses. Global multiclass performance indices (accuracy, *κ*, balanced accuracy, macro-F1) are summarised in Table 5.

**Table 3:** Confusion matrix: ChatGPT-predicted versus EPS-confirmed ventricular ectopy localisation.

Actual/Predicted	RVOT	LVOT	Papillary	Fascicular	Epicardial	Row total
RVOT (*n* = 30)	**12**	12	4	0	2	30
LVOT (*n* = 11)	5	**4**	1	0	1	11
Papillary (*n* = 4)	1	1	**1**	0	1	4
Fascicular (*n* = 1)	1	0	0	**0**	0	1
Epicardial (*n* = 4)	2	2	0	0	**0**	4
Column total	21	19	6	0	4	**50**

Correct predictions (bold): 17/50—overall accuracy of 34%.

**Table 4:** Diagnostic performance of ChatGPT by anatomical region.

Region	Sensitivity, %	Specificity, %	PPV, %	NPV, %
RVOT	40	55	57	38
LVOT	36	62	21	77
Papillary muscle	25	89	17	93
Fascicular	0	100	–	98
Epicardial/LV-summit	0	91	0	91
Overall	–	–	–	–

PPV = positive predictive value; NPV = negative predictive value; “–” indicates not calculable because no positive predictions were issued for that category.

**Table 5:** Non-invasive localisation versus EAM (gold standard): paired comparison of electrophysiologist (EP) and ChatGPT, global performance vs. EAM.

Metric	Electrophysiologist (EP)	ChatGPT
Overall accuracy, % (95% CI)	**72.0** (≈ 59–82)*	**34.0** (≈ 22–48)†
Cohen’s κ (95% CI)	**0.52** (≈ 0.36–0.70)*	**−0.02** (−0.18 to 0.14)
Balanced accuracy, 5-class macro-recall, %	69.9*	**20.2**
Macro-F1 (5-class)	0.71*	**0.19**
Outflow vs. non-outflow accuracy, %	88.0*	**70.0** (TP = 33, TN = 2, FP = 7, FN = 8)
Outflow sensitivity / Non-outflow specificity, %	95.1/55.6*	**80.5/22.2**

Primary endpoint: paired accuracy difference (McNemar). Secondary: Cochen’s κ vs. EAM, balanced accuracy, macro-F1, and coarse outflow/non-outflow metrics. Primary paired comparison (EP vs. ChatGPT, 5-class): Δaccuracy (EP–ChatGPT) = +38 pp (bootstrap 95% CI ≈ + 22 to +53)*, McNemar *p* < 0.001 (paired). †Indicates a statistically significant difference compared with the electrophysiologist based on the primary paired analysis (McNemar test, *p* < 0.001). Bold values indicate correct predictions and/or primary performance metrics.

Performance did not differ significantly between patients with SHD (3/12 correct, 25%) and those without SHD (14/38 correct, 37%) (χ^2^ = 0.63, *p* = 0.43). Likewise, age, PVC burden, and QRS duration were comparable between correctly and incorrectly classified cases (all *p* > 0.20; data not shown). Procedure duration (119 ± 23 min vs. 122 ± 27 min, *p* = 0.61) and acute ablation success (94% vs. 97%, *p* = 0.59) were similar regardless of ChatGPT accuracy, indicating that, within this cohort, the AI model’s predictions would not have altered immediate clinical outcomes. Collectively, these findings demonstrate that text-prompted ChatGPT reasoning failed to achieve clinically meaningful agreement with invasive mapping and was particularly unreliable for non-outflow-tract ventricular ectopy.

In a blinded analysis, the EP achieved 72.0% overall accuracy (95% CI 59–82) with moderate agreement (*κ* = 0.52, 95% CI 0.36–0.70) relative to the EAM gold standard, versus 34.0% (95% CI 22–48) and Cohen’s κ = −0.02 (95% CI –0.18–0.14) for ChatGPT. The paired accuracy difference was +38 pp. (bootstrap 95% CI, +22 to +53; McNemar *p* < 0.001). EP gains were concentrated in RVOT (sens/spec 76.7/75.0%) and LVOT (72.7/82.1%); non-outflow recognition remained challenging for both methods, but favoured the EP (outflow vs. non-outflow accuracy 88.0%; sens/spec 95.1/55.6%) compared with ChatGPT (70.0%, 80.5/22.2%). See Tables 1, 5, 6 for a full summary and case-level cross-classification.

**Table 6:** Regional diagnostic performance (vs. EAM).

Region	EP Sens %	EP Spec %	EP PPV %	EP NPV %	ChatGPT Sens %	ChatGPT Spec %	ChatGPT PPV %	ChatGPT NPV %
RVOT	**76.7**	**75.0**	**82.1**	**68.2**	40	55	57	38
LVOT	**72.7**	**82.1**	**53.3**	**91.4**	36	62	21	77
Papillary	**50.0**	**97.8**	**66.7**	**95.7**	25	89	17	93
Fascicular	**100**	**100**	**100**	**100**	0	100	–	98
Epicardial/LV summit	**50.0**	**97.8**	**66.7**	**95.7**	0	91	0	91

Bold values indicate correct predictions and/or primary performance metrics.

## Discussion

The precise preprocedural localisation of ventricular ectopic foci is critically important for successful ablation of VEs. Accurate prediction of the origin of a VEs from a 12-lead electrocardiogram (ECG) can guide the operator’s approach (e.g., determining whether the focus lies in the right ventricular outflow tract (RVOT) versus the left ventricular outflow tract (LVOT)), thereby informing the vascular access route and potentially shortening the procedure time (1). In contrast, failing to correctly localise the site of origin (SOO) beforehand may lead to prolonged mapping in the wrong cardiac region, increased fluoroscopy exposure, higher complication risk, and ultimately a lower ablation success rate (9). Thus, for electrophysiologists, having a reliable estimate of the VEs origin is invaluable in planning and executing catheter ablation efficiently and safely (10).

Several methods have been developed over the years to estimate the localisation of idiopathic VEs (or premature ventricular contractions) prior to ablation. Conventional approaches rely on surface ECG criteria and algorithms. For example, various metrics based on QRS morphology in specific leads—such as the R-wave duration or amplitude indices in leads V₁–V₂, and the precordial transition (deflection) pattern—have been proposed to distinguish RVOT from LVOT origins (10). A “transition zone index” was even introduced to adjust for differences in cardiac rotation between patients. Among the best-performing classical criteria, Yoshida et al.’s “cardiac rotation-corrected” transitional zone (TZ) index differentiated RVOT from aortic sinus cusp ventricular arrhythmias with 88% sensitivity and 82% specificity (area under the curve 0.90) in a 112-patient cohort (11). Likewise, Betensky et al.’s V2 transition ratio, in a prospective series of outflow-tract PVCs/VT with V3 precordial transition, predicted an LVOT origin with ≈95% sensitivity, 100% specificity and ≈91% accuracy when the ratio was <0.60 (12). These quantitative benchmarks underscore that, even before the advent of modern deep-learning models, carefully engineered ECG criteria could achieve high accuracy for selected outflow-tract scenarios, whereas in our study, ChatGPT reached only 70% outflow vs. non-outflow accuracy and 20.2% balanced accuracy across five classes (12, 13). However, these rule-based algorithms have shown only moderate accuracy. Their performance often drops, especially in intermediate cases (e.g., when the QRS transition occurs around lead V₃), with reported accuracies well below 80% in such scenarios (14). In fact, one analysis of multiple ECG algorithms found that none exceeded 80% accuracy in localising outflow tract PVCs with mid-precordial transition, underscoring the inherent limitations of these methods (15). Moreover, many of these algorithms require cumbersome manual measurements (such as calculating amplitude ratios or indices not readily available from standard ECG software), which discourages their routine clinical use by operators (16). To overcome these issues, researchers have turned to advanced techniques. Non-invasive electrocardiographic mapping systems and artificial intelligence (AI) models are being explored to improve localisation precision. Notably, machine learning approaches that automatically extract ECG features have demonstrated promising results. For instance, the AI-enabled algorithm reported by Nakamura et al. could predict PVC origins with a weighted accuracy of approximately 85%, outperforming experienced electrophysiologists (who achieved approximately 73% accuracy) and approaching the success of the best traditional algorithm (17). Similarly, other groups have developed multi-stage classification models that hierarchically categorise ectopic ventricular arrhythmia origins—from broad regions down to specific anatomical sites—yielding higher precision than earlier methods (16). These emerging techniques illustrate that leveraging computational power and data-driven algorithms can enhance our ability to localise VE foci prior to ablation.

From a quantitative standpoint, our findings should be interpreted in light of prior AI-based localisation work. Artificial intelligence-enabled ECG algorithms that operate directly on digital waveforms or images have already achieved substantially higher performance than that observed with ChatGPT. For example, support vector machine and convolutional neural network models developed by Nakamura et al. reached weighted accuracies of 0.85 and 0.80, respectively, for four-class PVC origin classification, whereas board-certified electrophysiologists achieved 0.73 and the best rule-based algorithm 0.86 (17). A deep-learning algorithm by Chang et al. reported an AUC of 0.963 for left-versus-right ventricular origin and 0.998 for LV summit prediction, with sensitivities of 90.7–100% and specificities of 92.3–98% in a 731-patient cohort (6). Similarly, a high-precision machine learning system described by Zheng et al. achieved accuracies of 98–99%, F1 scores of approximately 98%, and balanced accuracies near 98% across up to 21 distinct anatomical sites (16). In contrast, the general-purpose language model evaluated in our study reached only 34% overall accuracy, 20.2% balanced accuracy, and a macro-F1 of 0.19 for five-way localisation, with Cohen’s *κ* ≈ −0.02 versus electroanatomical mapping, confirming that current conversational LLMs fall far short of state-of-the-art, task-specific localisation AI. Looking ahead, however, emerging multimodal transformer architectures—such as biomedical GPT models such as BioGPT and BioGPT-X (18), vision–language ECG models such as ECG-GPT (19), and medical adaptations of GPT-4 V (sometimes referred to as “Med-GPT4V”) including (20)—illustrate how domain-specific or vision–language systems could eventually integrate raw ECG signals, ECG images, and textual context in a single model. This trajectory is consistent with the perspective articulated by Cersosimo et al., who, in a dialogue-based exploration of AI in cardiology and electrophysiology, argue that large models such as ChatGPT are most promising when layered on top of dedicated signal-processing AI and mapping tools rather than used in isolation (7).

In parallel with these domain-specific tools, there is growing interest in harnessing general-purpose AI, such as ChatGPT, in healthcare, given its speed, accessibility, and cost-free availability. Recent studies suggest that large language models (LLMs) can function as decision support aids in cardiology. For example, ChatGPT has shown a remarkable capacity to answer clinical questions about acute coronary syndromes (ACS) accurately. In one evaluation, the chatbot provided correct and “highest quality” answers to over 90% of frequently asked questions about ACS, including management- and guidelines-based queries (21). Its responses on ACS topics were not only highly accurate but also consistent upon repetition, with nearly 94% reproducibility in answers—highlighting reliability in addition to correctness (21). Similarly, ChatGPT and related models have demonstrated proficiency in interpreting cardiac rhythm disturbances. In a proof-of-concept study focusing on arrhythmia detection, a GPT-4–based model achieved a sensitivity of 93% and specificity of 89% for identifying common rhythm disorders, including malignant arrhythmias such as ventricular tachycardia (22). The model’s diagnostic performance for arrhythmias was comparable to that of expert clinicians, and it even showed particular strength in recognising less common but life-threatening rhythms such as ventricular flutter/tachycardia (22). These findings underscore the potential of user-friendly AI applications, such as ChatGPT, to assist in acute cardiac care by rapidly synthesising information and offering interpretations that align closely with clinical truth.

Despite these successes in other domains, our study observed that ChatGPT’s performance in Ves’ localisation estimation was rather poor. There are several plausible reasons for this discrepancy. First, the task of pinpointing an arrhythmic focus from a surface ECG is inherently complex and fraught with nuances that a general language model may not capture. Small variations in heart anatomy or lead placement can significantly alter ECG morphology; for instance, even a subtle misplacement of precordial electrodes can shift the R-wave transition and mislead the predicted origin (12, 23). Additionally, VEs originating from certain locations (such as the LVOT) may occur along unusual pathways and produce ECG patterns mimicking a different site (such as the RVOT), as documented by Yamada et al. (15). These physiological quirks have historically caused traditional algorithmic predictions to falter, and a general AI lacking specialised training would be prone to the same pitfalls. Second, ChatGPT is fundamentally a language-based model without the inherent capability to perform quantitative signal analysis. It relies on learnt associations and text-based patterns rather than direct interpretation of electrogram data. Thus, when tasked with localisation, it likely draws on simplified heuristics or incomplete knowledge gleaned from its training data (for example, known ECG criteria from the literature) without the precision of a purpose-built mapping algorithm. Indeed, recent evaluations of ChatGPT-4 in handling raw ECG interpretation found that it often failed to accurately identify complex arrhythmias or ischaemic changes, with one report noting only 24% accuracy for the primary diagnosis in a set of diverse ECGs (22). This emphasises that while LLMs can converse about medical topics, they are not yet adept at the kind of detailed waveform analysis and nuanced pattern recognition that VE localisation demands. In our case, ChatGPT’s underwhelming results likely reflect these limitations—the model was essentially attempting a highly specialised ECG inference task with a generalist’s toolbox.

To contextualise the LLM’s performance with a human non-invasive benchmark, we added a blinded electrophysiologist (EP) comparator using the same five-region schema (RVOT, LVOT, papillary muscle, fascicular/His–Purkinje, or epicardial/LV summit). As shown in Tables 1, 5, 6, EP achieved 72.0% overall accuracy (95% CI 59–82) with moderate agreement (*κ* = 0.52, 95% CI 0.36–0.70), significantly higher than ChatGPT’s 34.0% (95% CI 22–48, κ = −0.02, 95% CI –0.18–0.14), with a paired accuracy difference of +38 pp. (McNemar *p* < 0.001). Improvements were most evident for RVOT/LVOT discrimination and recognition of non-outflow origins; epicardial/LV-summit foci remained challenging for both approaches.

Finally, it is important to contextualise our findings and consider the path forward. Our investigation highlights that, at present, an off-the-shelf conversational AI such as ChatGPT cannot replace dedicated mapping techniques or expert analysis for localising ectopic ventricular foci. The convenience and speed of ChatGPT’s free-text consultation come at the cost of domain-specific accuracy in this scenario. However, this does not diminish the broader potential of integrating such AI tools into electrophysiological workflows in the future. With further refinement—including training on larger datasets of arrhythmia cases, incorporation of real ECG signal input, and development of specialised model architectures—AI assistants might substantially improve tasks such as localisation estimation. At a technical level, meaningful gains will likely require (i) domain-specific fine-tuning of large models on sizeable VE ablation datasets that pair 12-lead ECGs with electroanatomical mapping labels; (ii) multimodal architectures in which raw ECG signals (or ECG images), structured clinical features (e.g., age, QRS duration, presence of structural heart disease), and textual descriptors are jointly encoded and fed into a language model; and (iii) tight coupling between a conversational interface and task-optimised ECG encoders or mapping algorithms, so that the LLM acts as a user-friendly front end rather than as the sole diagnostic engine. Recent reviews stress that improving the quality of training data and creating more domain-focused versions of models will be crucial to enhancing the clinical utility of LLMs (24). In conclusion, although the current performance of ChatGPT in VE ablation planning is suboptimal, it represents a starting point for innovation. Going forward, a synergistic approach that combines the rapid information processing of ChatGPT with the rigour of validated electrophysiological algorithms may eventually yield a powerful decision-support system. From an ethical perspective, our results also highlight the risks of automation bias and over-reliance on generative AI. In this cohort, the ChatGPT frequently produced confident and fluent explanations despite a good level of agreement with the invasive reference standard (*κ* ≈ 0), which could anchor clinical decisions if used without oversight. Consistent with recent commentaries on ChatGPT in cardiology and electrophysiology, any deployment of such tools should ensure explicit human supervision, transparent communication of model limitations and training data, and clear institutional governance regarding accountability and data protection (7, 24). Until then, clinicians should remain cautious, using such tools as adjuncts—not substitutes—and continue to rely on established mapping methods and clinical judgement for critical localisation decisions (24).

### Limitations

Several constraints should be acknowledged when interpreting the findings of this study. First, this was a single-centre study with a relatively small cohort (*n* = 50), and RVOT/LVOT origins accounted for more than four-fifths of all ventricular ectopic (VE) foci. The narrow case mix and limited sample size reduce the statistical power for less common sites (papillary muscles, fascicular, and epicardial foci) and limit generalisability to centres that treat a broader spectrum of idiopathic VEs. Accordingly, our work should be interpreted as an exploratory feasibility pilot study rather than as a definitive evaluation of the performance of ChatGPT across all VE substrates.

Second, patients whose clinical VE could not be induced or precisely mapped were excluded, and those with multifocal VEs were not studied. These exclusions introduce selection bias towards morphologies that are inherently easier to locate and ablate, potentially inflating or deflating ChatGPT’s apparent performance relative to routine clinical practice.

Third, all ECG text descriptors were created by a single senior electrophysiologist, and no inter-observer reproducibility study was performed. Variability in how clinicians phrase morphological features could materially affect the interpretation of ChatGPT, and our workflow does not capture this uncertainty. Likewise, ECGs were recorded up to 30 days before ablation; spontaneous changes in the coupling interval or conduction could alter the QRS morphology between recording and procedure. In addition, we did not perform a formal assessment of intra-procedural reproducibility or inter-operator agreement for electroanatomical mapping. Invasive maps were created and interpreted according to routine clinical workflow at a single tertiary centre, and the final site of origin was defined by activation/pacemapping criteria together with acute VE elimination. Although this composite endpoint is widely accepted in idiopathic VE ablation, small errors in labelling the reference standard cannot be excluded, and our results should be interpreted in that context.

Fourth, the study evaluated only one prompt structure, a single model iteration, and fixed decoding parameters (ChatGPT 4o, March 2025 snapshot; temperature = 0.2, max tokens = 128). Large language models are highly sensitive to prompt engineering, sampling temperature, and continuous model updates. We did not perform a systematic hyperparameter search across alternative temperatures or token limits, nor did we systematically repeat queries for each case to characterise the within-case output variability under the chosen configuration. Different prompts (e.g., stepwise reasoning chains or ensemble querying), parameterisations, or future model versions might perform differently, so our results should not be extrapolated to all possible ChatGPT configurations.

Fifth, we did not reimplement established ECG-based localisation algorithms (such as the Yoshida transitional zone index or the Betensky V2 transition ratio) or other bespoke AI tools on our own dataset, nor did we quantify the incremental value of incorporating demographic or imaging data. Our comparisons with rule-based and machine-learning systems are therefore drawn from published performance metrics rather than from head-to-head testing on the same cohort. Retrospective application of these criteria would require additional manual measurements and is particularly sensitive to outflow-tract-specific inclusion criteria (e.g., V3 precordial transition), which our small pilot sample is underpowered to support. Future studies with larger, multicentre datasets should include direct algorithmic benchmarks and multimodal models to more precisely position general-purpose LLMs within the existing ECG localisation landscape.

Collectively, these limitations highlight the need for larger, multi-centre, multi-modal studies with rigorous comparative arms before definitive conclusions can be drawn about the clinical utility of general-purpose language models in VE localisation.

## Data Availability

The raw data supporting the conclusions of this article will be made available by the authors without undue reservation.
