# Supplementary material for: Diagnostic accuracy of ChatGPT for 12-lead ECG-based localisation of ventricular ectopic foci prior to catheter ablation
Source: Front Med (Lausanne). 2026 Jan 12;12:1685419. doi: 10.3389/fmed.2025.1685419 (PMC12833440; doi:10.3389/fmed.2025.1685419)
Supplement: Supplementary file 1 [file Table_1.docx]

**Supplementary Table S1. Example 12‑lead ECG textual descriptions and ChatGPT prompt**

This table provides representative examples of the textual ECG descriptions used as input for ChatGPT, along with the exact prompt submitted to the model. Each description was anonymized and structured according to the rule‑based template applied throughout the study (axis, bundle‑branch pattern, precordial transition, notching/slurring, concordance, and other salient features).

| Case ID | Structured ECG Text Description | ChatGPT Prompt |
| --- | --- | --- |
| S01 | Frontal QRS axis: inferior; Bundle branch pattern: LBBB-like; Precordial transition: V3; Notching: absent; Limb-lead concordance: positive in inferior leads; R/S ratio > 1 in V4–V6. | Based on the following 12‑lead ECG features, determine the most likely anatomical origin of the ventricular ectopy: [Frontal QRS axis: inferior; Bundle branch pattern: LBBB-like; Precordial transition: V3; Notching: absent; Limb-lead concordance: positive in inferior leads; R/S ratio > 1 in V4–V6.] Choose one: RVOT, LVOT, papillary muscle, fascicular/His–Purkinje, epicardial/LV summit. |
| S14 | Frontal QRS axis: superior; Bundle branch pattern: RBBB-like; Precordial transition: V5; Notching: subtle notching in V2–V3; Limb-lead concordance: negative in inferior leads. | Based on the following 12‑lead ECG features, determine the most likely anatomical origin of the ventricular ectopy: [Frontal QRS axis: superior; Bundle branch pattern: RBBB-like; Precordial transition: V5; Notching: subtle notching in V2–V3; Limb-lead concordance: negative in inferior leads.] Choose one: RVOT, LVOT, papillary muscle, fascicular/His–Purkinje, epicardial/LV summit. |
| S27 | Frontal QRS axis: leftward; Bundle branch pattern: LBBB-like; Precordial transition: V2; Notching: marked slurring in V1–V3; Limb-lead concordance: mixed; QRS duration: 160 ms. | Based on the following 12‑lead ECG features, determine the most likely anatomical origin of the ventricular ectopy: [Frontal QRS axis: leftward; Bundle branch pattern: LBBB-like; Precordial transition: V2; Notching: marked slurring in V1–V3; Limb-lead concordance: mixed; QRS duration: 160 ms.] Choose one: RVOT, LVOT, papillary muscle, fascicular/His–Purkinje, epicardial/LV summit. |
| S39 | Frontal QRS axis: rightward; Bundle branch pattern: RBBB-like; Precordial transition: V4; Notching: absent; Limb-lead concordance: positive in leads II, III, aVF; Deep S in I, aVL. | Based on the following 12‑lead ECG features, determine the most likely anatomical origin of the ventricular ectopy: [Frontal QRS axis: rightward; Bundle branch pattern: RBBB-like; Precordial transition: V4; Notching: absent; Limb-lead concordance: positive in leads II, III, aVF; Deep S in I, aVL.] Choose one: RVOT, LVOT, papillary muscle, fascicular/His–Purkinje, epicardial/LV summit. |
| S45 | Frontal QRS axis: inferior; Bundle branch pattern: LBBB-like; Precordial transition: V1; Notching: distinct pseudo‑delta wave; Limb-lead concordance: negative; Prolonged QRS (170 ms). | Based on the following 12‑lead ECG features, determine the most likely anatomical origin of the ventricular ectopy: [Frontal QRS axis: inferior; Bundle branch pattern: LBBB-like; Precordial transition: V1; Notching: distinct pseudo‑delta wave; Limb-lead concordance: negative; Prolonged QRS (170 ms).] Choose one: RVOT, LVOT, papillary muscle, fascicular/His–Purkinje, epicardial/LV summit. |
